# New Brain Aging Center

**DOI:** 10.1093/geroni/igab046.779

**Published:** 2021-12-17

**Authors:** Feng Lin, Yeates Conwell, Janine Simmons

## Abstract

Evidence indicates an association between emotional well-being (EWB) and underlying brain processes, and that those processes change with both normal and pathological brain aging. However, the nature of these associations, the mechanisms by which EWB and its component domains change with brain aging, and how those changes may be associated with common neuropathologies like Alzheimer’s disease and related dementias (ADRD), are largely unexplored. The NIA-funded Network for Emotional Well-being and Brain Aging (NEW Brain Aging) has the goal of developing a nationwide community of investigators dedicated to research that identifies and tests mechanisms by which brain aging influences EWB and how EWB may impact risk for and progression of ADRD. Synthesizing human and animal literature, our premise is that relationships between EWB and ADRD are bidirectional – normal and pathological changes in aging brain influence EWB and EWB contributes to brain health and illness, such as ADRD. NEW Brain Aging will identify and coalesce resources for interested investigators and provide pilot funding opportunities to stimulate research and development of the field. Component presentations of this symposium will include (1) an overview by Dr. Robert Kaplan of the current state of research on EWB; (2) the role of animal studies (Kuan Hong Wang) and (3) human subjects research (Feng Vankee Lin) in EWB and aging; and (4) design of NEW Brain Aging and resources it will provide (Yeates Conwell). Janine Simmons will explain NIA’s vision for EWB research and lead open discussion.

